# Octylisothiazolinone—A New Sensitizer in Over‐Ear Headphones

**DOI:** 10.1111/cod.70028

**Published:** 2025-09-13

**Authors:** Blerand Berisha, Tina Lejding, Ola Bergendorff, Inese Hauksson

**Affiliations:** ^1^ Department of Dermatology Helsingborg Hospital Helsingborg Sweden; ^2^ Department of Occupational and Environmental Dermatology Lund University, Skåne University Hospital Malmö Sweden

**Keywords:** allergic contact dermatitis, octylisothiazolinone, over‐ear headphones, patch test

## Abstract

**Background:**

Allergic contact dermatitis (ACD) caused by isothiazolinones is a growing concern, particularly in consumer products. Octylisothiazolinone (OIT) is a potent sensitiser and commonly used as a preservative in rubbers, plastics and coatings, including those found in headphones. Despite increasing regulations on isothiazolinones in cosmetics, their presence in wearable devices remains under‐recognised.

**Aims:**

This investigation aims to identify OIT as a possible sensitiser in patients with presumed ACD due to over‐ear headphones.

**Methods:**

Two patients with presumed ACD due to over‐ear headphones were patch tested at the Department of Occupational and Environmental Dermatology, Skåne University Hospital, Malmö. Patch testing included Swedish baseline series, isothiazolinone compounds, and materials from headphones. To identify potential allergens, chemical analysis of components from headphones was conducted using high‐performance liquid chromatography (HPLC).

**Results:**

Patch testing demonstrated strong positive reactions to OIT in both cases, with additional reactions to acetone extracts of artificial leather and blue plastic foam. OIT remained positive at dilutions as low as 0.00003%. Case 1 also showed positive reactions to MI/MCI. Chemical analysis confirmed OIT in both artificial leather (2.2 mg/g) and plastic foam (0.3 mg/g).

**Conclusions:**

These findings contribute to the understanding of ACD by identifying OIT as a potential allergen in over‐ear headphones. It calls for further research into the prevalence of OIT in consumer electronics and its role in sensitisation.

## Graphical abstract

Two cases of allergic contact dermatitis from over‐ear headphones are presented. OIT was confirmed by chemical analysis in one case and implicated by patch testing in the other, underscoring its relevance as a sensitiser in consumer products.

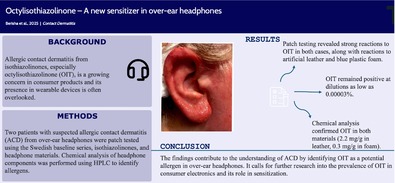

## 1 Introduction

Isothiazolinones, including methylchloroisothiazolinone (MCI), benzisothiazolinone (BIT), and octylisothiazolinone (OIT), have recently been identified as significant causes of allergic contact dermatitis (ACD), particularly in patients using headphones [1]. These organic compounds are commonly utilised as preservatives or biocides due to their potent antimicrobial and fungiostatic properties [2]. Isothiazolinones are widely incorporated into industrial, household, and cosmetic products, where they offer effective microbial control but also present a notable risk of sensitisation. ACD due to isothiazolinones is a delayed‐type hypersensitivity reaction mediated by antigen‐specific T cells which recognise the allergen and start an inflammatory response upon re‐exposure [3]. The increasing use of isothiazolinones, largely driven by their effectiveness, has been paralleled by growing concern regarding their sensitisation potential, leading to numerous cases of ACD [2, 4]. OIT, in particular, is frequently used as a preservative in various products such as water‐based paints, detergents, cooling fluids, and leather. In recent years, several reports have emerged documenting cases of ACD linked to OIT exposure, particularly through leather goods [5, 6, 7]. Similarly, cases of ACD related to wireless headphones have been reported, with known allergens including gold and acrylates being implicated in reactions associated with in‐ear headphones [8, 9, 10, 11]. However, reports of ACD linked to over‐ear headphones are relatively rare. To date, there have been several cases that have identified isothiazolinones as the allergen responsible [1, 12]. In this report, we present two patients who developed ACD as a result of exposure to over‐ear headphones. These cases underscore the potential for isothiazolinones, specifically OIT, to provoke allergic contact dermatitis through unexpected sources, such as consumer electronic devices, warranting further investigation into their use in non‐traditional applications.

## 2 Patients and Methods

### 2.1 Patients

#### 2.1.1 Case 1

**FIGURE 1:**
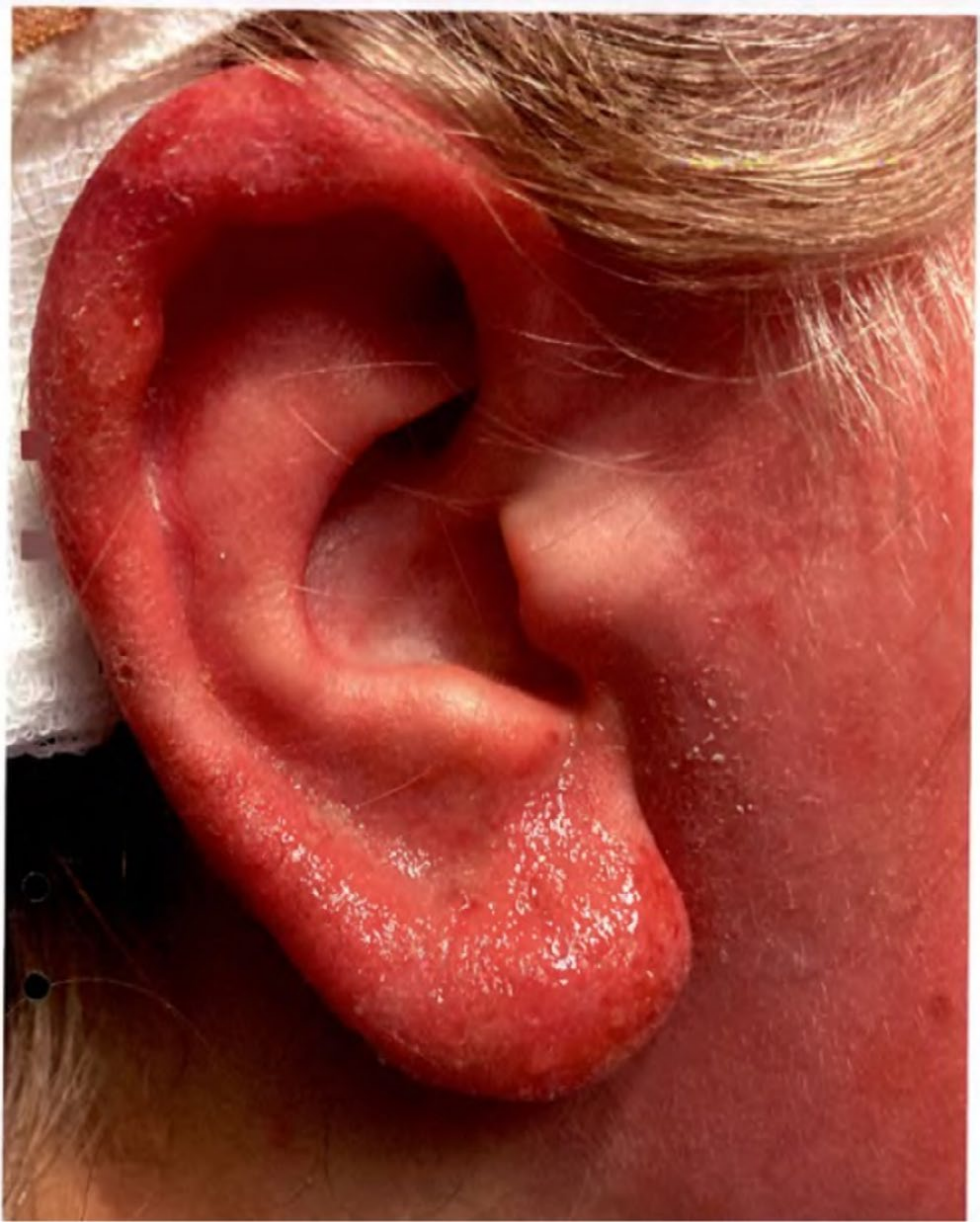
Erythematous oozing erosions and swelling presented on the ear of Case 1. May 2023.

Case 1 was an 11‐year‐old girl with no previous skin disease or allergy. Due to ADHD, she had treatment with methylfenidate and aiding tools such as headphones, which she used regularly in school and at home to help with her attention deficit. In January 2023, she presented with erythematous oozing erosions on her ears and swelling stretching all the way to her cheeks (Figure 1). It was initially diagnosed as bilateral erysipelas, and she was admitted to an ENT ward for treatment with IV antibiotics. She was, however, afebrile, and blood samples were normal. She had used over‐ear headphones both at school and at home, including a new pair received in December 2022. A few days after starting use, she developed dermatitis. ACD was suspected, and she was advised to stop the usage of the headphones. After treatment with topical steroids and resolution, she used another pair of headphones with faux leather, which again resulted in dermatitis, prompting a new course of treatment. Besides this, she had used headphones that belonged to her school with an inner coating of faux fur, which had caused pruritus but no dermatitis. She was referred to the Department of Occupational and Environmental Dermatology, Skåne University Hospital, Malmö for further investigation with patch testing.

#### 2.1.2 Case 2

**FIGURE 2:**
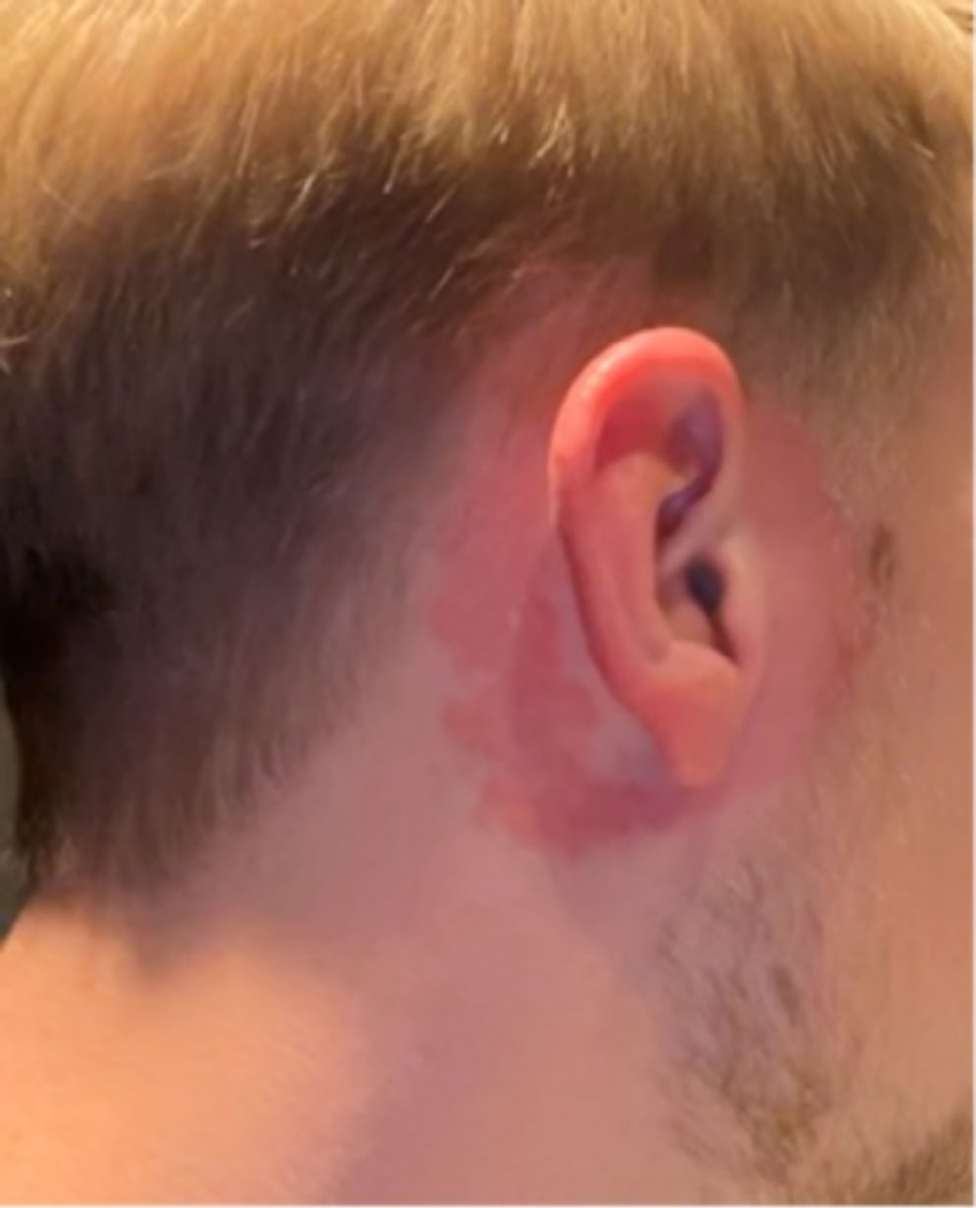
Erythematous dermatitis presented on and around the ear of Case 2, 23 August 2023.

Case 2 was a 22‐year‐old man with no previous skin disease. He was allergic to fur‐bearing animals. He had received over‐ear headphones for Christmas and used these for less than 1 month before he developed an itching dermatitis on and around the ears (Figure 2). He had tried to clean the headphones and also ordered new ones in order to use them, although always with a subsequent dermatitis. Once he had the headphones on his chin, he also developed itching but no dermatitis. Upon discontinued use of the headphones, the dermatitis resolved with topical steroid treatment within a week. On suspicion of ACD, he was referred to the Department of Occupational and Environmental Dermatology, Skåne University Hospital, Malmö for further investigation with patch testing.

### 2.2 Patch Testing

Cases 1 and 2 were investigated at the Department of Occupational and Environmental Dermatology, Skåne University Hospital, Malmö. All patch testing preparations used were from Chemotechnique Diagnostics (Vellinge, Sweden). Testing was performed with occlusion of IQ Ultra chambers (SmartPractice, Phoenix, Arizona) in both cases. Patches were occluded on the back of the patients with subsequent physician assessment on day (D) 3 and D7. Scoring based on reaction was performed in accordance with the ICDRG and ESCD criteria [13, 14].

**FIGURE 3:**
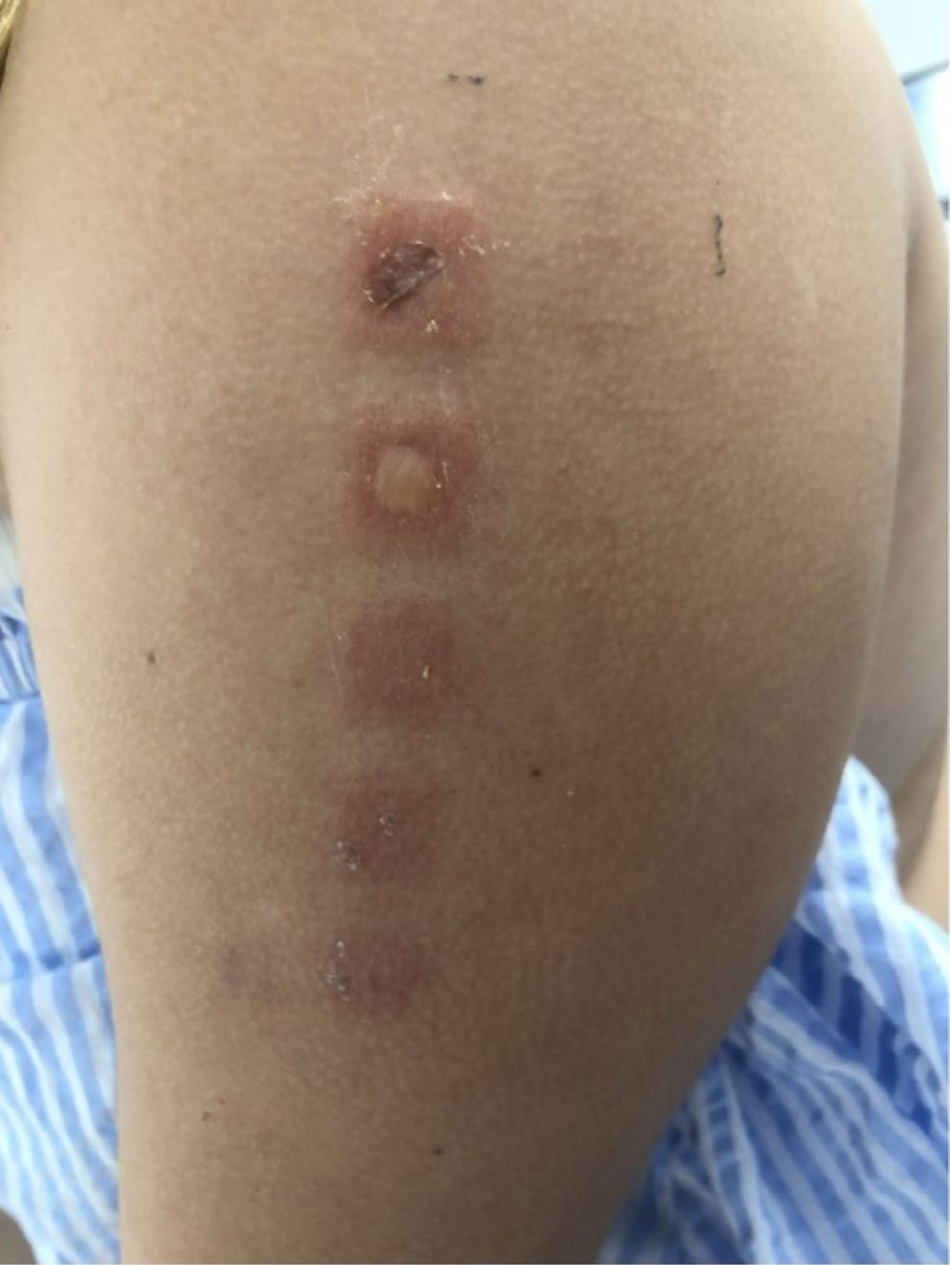
Patch testing result for OIT in dilution in Case 1. Written informed consent was obtained from the patient and their parents for the publication of this image. 26 May 2023.

Case 1 was tested with the revised Swedish baseline series for children, Isocyanate series, BIT and OIT with dilution. During the initial evaluation, the patient underwent patch testing using the Swedish baseline series for children, along with testing of her personal headphones. Notably, the tests revealed significant reactions to the headphones and methylisothiazolinone + methylchloroisothiazolinone (MI + MCI) in water. Given the strong response to MI + MCI, we extended our investigation to identify isothiazolinone‐like substances in the chemical composition of the headphones. This analysis led to the detection of octylisothiazolinone (OIT). Consequently, we proceeded with patch testing for OIT in dilutions made in alcohol to further assess its role in the patient's reaction (Figure 3).

Case 2 was tested with the Swedish baseline series for adults, an extended in‐house series, plastics series, textile series, and Oxidised Linalool and Limonene series. Both cases were tested with the artificial leather from their respective headphones. The artificial leather was tested as is in case 1, as is and in acetone ultrasonic extract in case 2. Case 1 was also tested with acetone ultrasonic extract made from a blue plastic foam and faux fur part of the headphones. The extracts were concentrated to a volume of 1 mL before evaporation with subsequent use for testing [15].

## 3 Results

### 3.1 Patch Test

The relevant patch test results are summarised in Table 1.

**TABLE 1:** Summary of patch test reactions in two patients who reacted positively to Octylisothiazolinone (OIT).

Test preparation	Test reactions in Case 1	Test Reactions in Case 2
Methylisothiazolinone + Methylchloroisothiazolinone (MI + MCI) 0.215%	**+++**	**−**
2‐n‐Octyl‐4‐isothiazolin‐3‐one 0.1%	NT	**+++**
2‐n‐Octyl‐4‐isothiazolin‐3‐one 0.01%	**+++**	NT
2‐n‐Octyl‐4‐isothiazolin‐3‐one 0.003%	**+++**	NT
2‐n‐Octyl‐4‐isothiazolin‐3‐one 0.001%	**+++**	NT
2‐n‐Octyl‐4‐isothiazolin‐3‐one 0.0003%	**+++**	NT
2‐n‐Octyl‐4‐isothiazolin‐3‐one 0.0001%	**+++**	NT
2‐n‐Octyl‐4‐isothiazolin‐3‐one 0.00003%	**+**	NT
2‐n‐Octyl‐4‐isothiazolin‐3‐one 0.00001%	**−**	NT
Benzisothiazolinone 0.1% pet	**−**	**−**
Benzisothiazolinone 0.15% pet	**−**	**−**
Black artificial leather^a^	**+++**	**+++**
Black artificial leather^b^	+++	**+++**
Blue plastic foam^a^	**+++**	NT
Faux fur^a^	?	NT

*Note*: +++ = Very strong response, ++ = Moderate response, + = Mild response, *−* = Negative reaction, ? = Doubtful reaction, NT = not tested.

^a^ Acetone extract.

^b^ As is.

In case 1, positive reactions in the Swedish baseline series for children were observed with +++ for MI + MCI. Positive reactions in the personal materials series were observed with +++ for acetone extract of artificial leather from headphones and plastic foam and artificial leather as is. OIT with further dilution from 0.01% to 0.00003% was positive. The faux fur extract was evaluated as a doubtful reaction.

In case 2, positive reactions in the Swedish baseline series add on for Malmö were observed with +++ for OIT. Positive reactions in the personal materials series were observed with +++ for artificial leather extract and as is. OIT with further dilution was not tested.

### 3.2 Chemical Investigations

**FIGURE 4:**
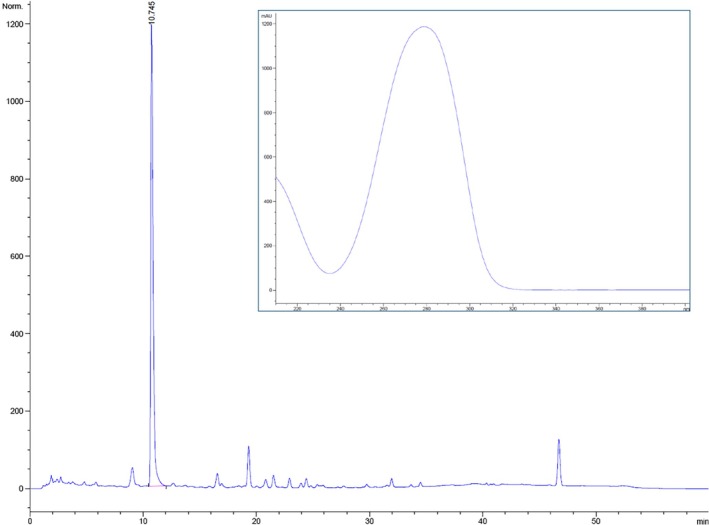
Chemical analysis of extract from black artificial leather sample from case 1. The chromatogram is showing octylisothiazolinone peak at 10.7 min. Ultraviolet spectrum from this peak is inserted. Apex in spectrum is at 280 nm and the spectrum is identical to the spectrum from a reference sample of octylisothiazolinone.

Both black artificial leather and blue plastic foam from the headphone of case no 1 were analysed with a high‐performance liquid chromatography (HPLC) method originally developed for identifying rubber allergens in medical gloves [16]. A reversed‐phase column (Alltima C18, 4 mm, 150 × 4.6 mm^2^, polyether ether ketone [PEEK]‐lined; Alltech Associates, Deerfield, IL, USA) was eluted with a gradient composed of acetonitrile and aqueous zinc sulphate (10^−5^ mol/L) at a ratio of 50:50 for 5 min, and then with a linear gradient to 100% acetonitrile for 35 min. The mobile phase was pumped with an Agilent 1260 (Agilent Technologies, Santa Clara, CA, USA) at a flow rate of 1 mL/min and monitored at 280 nm with an Agilent 1100 Series diode‐array detector (Agilent Technologies). Capillaries in contact with the eluent after the injector were made of PEEK. Peak area was used to determine the concentration, and the identification was made by comparison of retention time (10.7 min) and UV spectrum recorded by the diode‐array detector (Figure 4). A sample of OIT with known concentration was analysed and peak areas were compared with peaks from headphone samples. The concentrations were determined to be 2.2 mg/g OIT in the black artificial leather and 0.3 mg/g OIT in the blue plastic foam. No other isothiazolinone could be detected.

Chemical analysis was not performed in Case 2. This was primarily due to the fact that the connection between the two cases was recognised during the clinical investigation of Case 1. As the patients used headphones from the same brand and presented with similar clinical findings, it was assumed that the material composition was likely identical. Further chemical analysis of Case 2's headphones was discussed, but the patient declined extended evaluation due to lack of time related to occupational commitments.

## 4 Discussion

While ACD due to materials used in headphones is recognised, the identification of OIT as a sensitiser in over‐ear headphones introduces a new dimension to this issue.

Headphones, particularly over‐ear models, are becoming an emerging trigger factor for ACD due to the materials in contact with the skin, such as synthetic leathers and foams. These materials often create warm and humid environments, which can promote microbial growth. In this context, the use of antimicrobial agents like OIT, which may have been employed to mitigate microbial growth on surfaces, appears to have introduced an additional risk factor.

We believe that these cases had ACD explained by allergy to OIT, which was confirmed in Case 1. Further, MI + MCI were not detected in the headphones upon chemical analysis. Interestingly, Case 1 also reacted to MI + MCI, while Case 2 did not, raising the question of possible cross‐sensitisation or partial cross‐reactivity between isothiazolinones. This phenomenon, previously suggested in the literature, may be due to shared epitopes that elicit T‐cell‐mediated immune responses despite differences in molecular size or lipophilicity [17, 18]. Such immunological overlap could account for why Case 1 reacted to both MI/MCI and OIT, whereas Case 2 reacted only to OIT.

An important limitation is that no chemical analysis was performed in Case 2. While both patients used headphones from the same brand and presented with similar clinical findings, the absence of direct material testing in Case 2 limits the strength of casual link. However, given the materials in both cases were from the identical brand, similar exposure patterns, and comparable patch test reactivity to OIT, it remains plausible that the material composition was the same. To our knowledge, there has been no relapse of dermatitis after stopping the exposure to over‐ear headphones.

OIT has been well‐documented as a sensitizer in various consumer products [4, 5, 6]. However, its application in electronics, particularly in headphones, is less well known. The findings from this report suggest that the presence of OIT in the padding of the over‐ear headphones tested can contribute to sensitization and subsequent ACD in susceptible individuals. Our chemical investigation showed elevated concentrations of OIT found in the artificial black leather components of headphones compared to the blue foam part. This observation aligns with previous reports linking OIT in artificial leather products to ACD cases [12]. Similarly, a study reported ACD linked to OIT in leather sofas, emphasising the association between artificial leather components and OIT‐related ACD [19].

From a regulatory perspective, all chemical substances, including potential sensitizers like OIT, must comply with general EU REACH obligations [20]. However, OIT is as of date not listed among the substances subject to specific restrictions or authorisation requirements, and therefore may legally be used in consumer electronics such as headphone padding [21]. Its presence in such products suggests that certain applications, particularly in items with prolonged skin contact, may fall outside explicit regulatory control. This underscores the need for ongoing regulatory review to address potential gaps in consumer protection.

This study underscores the need for increased awareness among manufacturers and consumers regarding the use of chemical agents in consumer electronics. While the antimicrobial benefits of OIT are advantageous in preventing microbial contamination, its potential to induce ACD should not be overlooked. Furthermore, this report highlights the necessity for improved labeling and transparency about the chemical components used in electronic devices. Awareness of such allergens can help individuals with known sensitivities make informed choices and avoid products that may trigger allergic responses.

## 5 Conclusion

In conclusion, this study contributes to the understanding of ACD by identifying OIT as a potential allergen in over‐ear headphones. It calls for further research into the prevalence of OIT in consumer electronics and its role in sensitisation, as well as for regulatory bodies to ensure adequate control of sensitizers in consumer products.

## Author Contributions

**Blerand Berisha:** conceptualisation, data curation, formal analysis, validation, writing – original draft, writing – review and editing. **Tina Lejding:** supervision, data curation, writing – review and editing. **Ola Bergendorff:** investigation, supervision, methodology, validation, writing – review and editing. **Inese Hauksson:** conceptualisation, investigation, supervision, methodology, writing – review and editing.

## Conflicts of Interest

The authors declare no conflicts of interest.

## Data Availability

The data that support the findings of this study are available on request from the corresponding author. The data are not publicly available due to privacy or ethical restrictions.
